# Relationship between social development indicators and mortality due to Diabetes *Mellitus* in Brazil: a space-time analysis*

**DOI:** 10.1590/1518-8345.6592.3972

**Published:** 2023-08-14

**Authors:** Thiago Santos Garces, Lara Lídia Ventura Damasceno, George Jó Bezerra Sousa, Virna Ribeiro Feitosa Cestari, Maria Lúcia Duarte Pereira, Thereza Maria Magalhães Moreira

**Affiliations:** 1 Universidade Estadual do Ceará, Departamento de Enfermagem, Fortaleza, CE, Brasil.; 2 Becario de la Coordenação de Aperfeiçoamento de Pessoal de Nível Superior (CAPES), Brasil.; 3 Universidade Estadual do Ceará, Fortaleza, CE, Brasil.

**Keywords:** Diabetes Mellitus, Mortality, Social Indicators, Ecological Studies, Spatial Analysis, Time Series Studies, Diabetes Mellitus, Mortalidad, Indicadores Sociales, Estudios Ecológicos, Spatial Analysis, Estudios de Series Temporales, Diabetes Mellitus, Mortalidade, Indicadores Sociais, Estudos Ecológicos, Análise Espacial, Estudos de Séries Temporais

## Abstract

**Objective:**

to identify the space-time pattern of mortality due to Diabetes *Mellitus* in Brazil, as well as its relationship with social development indicators.

**Method:**

an ecological and time series nationwide study based on secondary data from the Unified Health System Informatics Department, with space-time analysis and inclusion of indicators in non-spatial and spatial regression models. The following was performed: overall mortality rate calculation; characterization of the sociodemographic and regional profiles of the death cases by means of descriptive and time analysis; and elaboration of thematic maps.

**Results:**

a total of 601,521 deaths related to Diabetes *Mellitus* were recorded in Brazil, representing a mean mortality rate of 29.5/100,000 inhabitants. The states of Rio Grande do Norte, Paraíba, Pernambuco, Alagoas and Sergipe, Rio de Janeiro, Paraná and Rio Grande do Sul presented high-high clusters. By using regression models, it was verified that the Gini index (β=11.7) and the Family Health Strategy coverage (β=3.9) were the indicators that most influenced mortality due to Diabetes *Mellitus* in Brazil.

**Conclusion:**

in Brazil, mortality due to Diabetes presents an overall increasing trend, revealing itself as strongly associated with places that have worse social indicators.

## Abstract (es)

**Objetivo:**

identificar el patrón espacial y temporal de la mortalidad por Diabetes *Mellitus* en Brasil y su relación con los indicadores de desarrollo social.

**Método:**

estudio ecológico y de series temporales, a nivel nacional, con base en datos secundarios del Departamento de Informática del Sistema Único de Salud, con análisis espacial y temporal e inserción de indicadores en modelos de regresión no espacial y espacial. Se realizaron: cálculo de la tasa de mortalidad general; caracterización del perfil sociodemográfico y regional de las muertes mediante análisis descriptivo y temporal; y elaboración de mapas temáticos.

**Resultados:**

en Brasil se registraron 601.521 muertes relacionadas con la Diabetes *Mellitus*, lo que representa una mortalidad media de 29,5/100.000 habitantes. Los estados de Rio Grande do Norte, Paraíba, Pernambuco, Alagoas y Sergipe, Río de Janeiro, Paraná y Rio Grande do Sul presentaron conglomerados alto-alto. Mediante el uso de modelos de regresión, se comprobó que el índice de Gini (β=11,7) y la cobertura de la Estrategia Salud de la Familia (β=3,9) fueron los indicadores que más influyeron en la mortalidad por Diabetes *Mellitus* en Brasil.

**Conclusión:**

la mortalidad por diabetes en Brasil tiene una tendencia general alcista, está fuertemente asociada a los lugares con peores indicadores sociales.

## Abstract (pt)

**Objetivo:**

identificar o padrão espacial e temporal da mortalidade por diabetes mellitus, no Brasil, e sua relação com indicadores de desenvolvimento social.

**Método:**

estudo ecológico e de séries temporais, de abrangência nacional, com base em dados secundários do Departamento de Informática do Sistema Único de Saúde, com análise espacial e temporal e inserção de indicadores em modelos de regressão não espacial e espacial. Realizaram-se: cálculo da taxa de mortalidade geral; caracterização do perfil sociodemográfico e regional dos óbitos mediante análise descritiva e temporal; e construção de mapas temáticos.

**Resultados:**

foram registrados 601.521 óbitos relacionados ao diabetes mellitus no Brasil, representando mortalidade média de 29,5/100.000 habitantes. Os estados do Rio Grande do Norte, Paraíba, Pernambuco, Alagoas e Sergipe, Rio de Janeiro, Paraná e Rio Grande do Sul apresentaram aglomerados alto-alto. Por meio do uso de modelos de regressão, verificou-se que o índice Gini (β=11,7) e a cobertura da Estratégia de Saúde da Família (β=3,9) foram os indicadores que mais influenciaram a mortalidade por diabetes mellitus no Brasil.

**Conclusão:**

a mortalidade por diabetes, no Brasil, exibe tendência geral ascendente, revelando-se fortemente associada a locais com piores indicadores sociais.

## Introduction

Diabetes *Mellitus* (DM) and its complications constitute a relevant and growing global public health problem, representing one of the leading causes of premature deaths in individuals over 60 years of age. Globally, recent data from the International Diabetes Federation show a total of 6.7 million deaths caused by the disease in 2021, reaching the milestone of one death every five seconds^(^ 1 ^)^.

Meanwhile, Brazil is the country with the highest number of people with Diabetes in Latin America, ranking fifth in the world. The mortality rates due to the disease have nearly doubled in the last decades, rising from 16.3 deaths *per* 100,000 inhabitants in 1996 to 29 deaths *per* 100,000 inhabitants in 2019, accounting for 30.1% of all premature deaths^(^ 2 ^)^.

The prevalence of Diabetes varies in the different Brazilian regions, with rates of 6.8% in the North, 8.7% in the Northeast, 10.5% in the Southeast, 8.5% in the South, and 10.3% in the Midwest. In this sense, the proportion of underreporting in the national scenario stands out, estimated at 72.8% in the North region^(^ 3 ^)^.

In light of this panorama, it is recognized that economic and social conditions decisively influence the quality of life and health of populations, while reflecting indicators such as wealth distribution, housing conditions, schooling and access to health services. Within the DM context, these indicators translate into lower knowledge about the disease, poorer clinical management quality and a higher risk of unfavorable outcomes such as acute and chronic complications or death^(^ 4 ^)^.

In this perspective, the literature reiterates the association of social determinants with hospitalizations^(^ 5 ^)^ and complications^(^ 6 ^)^ due to DM. However, there is lack of research studies evaluating these aspects as predictors of death due to DM. Therefore, it is reinforced that this is the first national study that strives to analyze the relationship between social development indicators and the outcome of death related to DM, also considering the effect of geographical space.

In addition, the study proposed is unique in its approach to guiding, planning and implementing Nursing care, given the understanding of the sociodemographic profile and behavioral trends of the outcome of death related to DM in the social, demographic and health environments. This study aims at favoring Nursing care qualification in a multiprofessional and interdisciplinary perspective, based on the (re)recognition of population demands, formulation and evaluation of interventions and prediction of future trends.

In this regard, the objective of this study was to identify the space-time pattern of mortality due to Diabetes *Mellitus* in Brazil and its relationship with social development indicators.

## Method

### Study design

An ecological study guided by the *Strengthening the Reporting of Observational Studies in Epidemiology* (STROBE) tool.

### Study locus

The study was conducted throughout the entire Brazilian territory (8,510,345.538 km²), distributed across five regions, 26 states and one Federal District (*Distrito Federal*, DF), considering its 5,570 municipalities as analysis units.

The geographic division of the country is done as follows in the following locations: 1) North Region - Acre (AC), Amapá (AP), Amazonas (AM), Pará (PA), Rondônia (RO), Roraima (RR) and Tocantins (TO); 2) Northeast Region - Alagoas (AL), Bahia (BA), Ceará (CE), Maranhão (MA), Paraíba (PB), Pernambuco (PE), Piauí (PI), Rio Grande do Norte (RN) and Sergipe (SE); 3) Southeast Region - Espírito Santo (ES), Minas Gerais (MG), Rio de Janeiro (RJ) and São Paulo (SP); 4) South Region - Paraná (PR), Rio Grande do Sul (RS) and Santa Catarina (SC); and 5) Midwest Region - Goiás (GO), Mato Grosso (MT), Mato Grosso do Sul (MS) and *Distrito Federal* (DF).

### Period

The study considered the available data from January 2010 to December 2020, while data collection and analysis were conducted from June 2021 to March 2022.

### Population definition

Deaths reported from January 2010 to December 2020 in individuals above ten years of age were included, related to the following categories from the Tenth International Classification of Diseases (ICD-10): E10 (Insulin-dependent DM), E11 (Non-insulin-dependent DM), E12 (Malnutrition-related DM), E13 (Other special types of DM), and E14 (Unspecified DM).

For this study, age groups starting from ten years old were considered, given the insignificant contribution of individuals under the age of ten to the outcome of death due to Diabetes *Mellitus*, which is reflected in the very low number of notifications in the national context, a fact supported by the literature^(^ 7 ^)^.

### Data collection

The secondary data were obtained from the website of the Unified Health System Informatics Department (DATASUS), specifically from the Vital Statistics section, which includes records of deaths by ICD-10, along with projections referring to the resident population (https://datasus.saude.gov.br/), in addition to Health Indicators and Agreements sourced from Administrative records and the 2010 *Atlas Brasil* Demographic Census (http://www.atlasbrasil.org.br/).

### Study variables

Regarding the predictive variables related to the health context and agreements, the following ones were selected: Gini index, *per capita* income, formalization percentage, *Bolsa Família* transfer, Social Vulnerability Index (SVI), Municipal Human Development Index (MHDI), population coverage percentage by Family Health Strategy teams, illiteracy in people aged over 18, percentage of households with a bathroom and running water, and percentage of population in households with density greater than two.

Selection of these variables was motivated by previous studies that performed space-time analyses^(^ 5 ^-^ 8 ^)^, reflecting characteristics related to demographics, schooling, income, employment and housing. It is also noted that the most recent data available in these databases were used. For instance, the primary care coverage databases are regularly updated on a monthly basis, whereas those related to the demographic census were last updated in 2010. However, these variables were still considered because they reflect the social, economic and health situation of the Brazilian population.

### Data analysis

Initially, the unadjusted mortality rate was calculated for each year. The calculation was based on the sum of the number of deaths during the period divided by the denominator of the population aged over ten years old and resident in each year, estimated by the Brazilian Institute of Geography and Statistics (*Instituto Brasileiro de Geografia e Estatística*, IBGE), and multiplied by the constant 100,000. In addition to that, a descriptive analysis was performed to characterize the sociodemographic profile of the sample regarding gender, age, schooling, race/skin color and region.

The annual time pattern of mortality due to Diabetes *Mellitus* in Brazil was evaluated, both overall and by regions and states, using line graphs generated in Microsoft Excel. The data were imported into the Joinpoint Regression software, version 4.6.0.0, for segmented linear analysis and visualization of the Annual Percentage Change (APC) and Average Annual Percentage Change (AAPC) with their 95% confidence intervals (CIs)^(^ 9 ^)^.

The analysis using joinpoints allows for the inclusion of multiple line segments, demonstrating changes in linear trends during the study period. It is noted that the connection between two straight line segments or two trends occurs at a joinpoint, which represents an inflection point. A significance level of p<0.05 was considered when the model showed statistical significance. It is important to note that positive and significant results indicate am increasing trend, while negative and significant results indicate a decreasing trend and non-significant results indicate a stationary pattern^(^ 9 ^)^.

For the spatial analysis, the mean rate was calculated by dividing the mean number of cases during the 11-year study period in each municipality by the estimated population in the middle of the period (2015). Finally, the result was multiplied by 100,000 inhabitants. Thematic maps were generated based on the mean unadjusted mortality rate during the entire study period. The rates were smoothed using the local empirical Bayesian method in order to reduce their instability. This method considers not only the value of the municipality but also weights it in relation to neighboring municipalities through a spatial proximity matrix. For calculation of the matrix, the contiguity criterion was used, assigning a value of 1 to municipalities that had neighboring municipalities and 0 to non-neighboring municipalities^(^ 10 ^)^.

The spatial autocorrelation of deaths was calculated using the Global Moran Index (GMI) based on the unadjusted indicators. The method identifies spatial autocorrelation and ranges from -1 to +1, where values close to zero indicate absence of spatial dependence. Once the GMI is found to be significant, the Local Indicators of Spatial Association (LISA) are calculated, evaluated by means of the Local Moran Index (LMI), enabling the identification of local patterns of deaths^(^ 11 ^)^.

In turn, LMI generates Moran scatterplots, which consist of four quadrants: high-high (cities with high rates surrounded by others with high rates), low-low (cities with low rates surrounded by others with low rates), high-low (cities with high rates surrounded by others with low rates), and low-high (cities with low rates surrounded by others with high rates). The “high-high” and “low-low” categories represent agreement areas, whereas “high-low” and “low-high” indicate epidemiological transition areas^(^ 12 ^)^.

In addition, in order to test the relationship of each socioeconomic indicator with the unadjusted mortality rate due to DM in Brazil, an univariate Ordinary Least Squares (OLS) regression analysis was performed. In this regard, the indicators were included in a non-spatial OLS “step forward” regression model. The variables that presented p<0.20 were included in a multivariate model considered final. After identifying the relationship between indicators and mortality due to DM, the hypothesis that this relationship also occurs in the geographic space was tested.

Therefore, those variables that remained in the final model were also included in a Geographically Weighted Regression (GWR) model, which takes into account the location of the municipalities when estimating their coefficients. To identify possible multicollinearity among the variables, the Variance Inflation Factor (VIF) was analyzed, where values above 10 indicate a violation of the assumption.

To select the model that best estimated this relationship, the GWR results were compared to OLS using two estimates: the R² value and the Akaike Information Criterion (AIC). The AIC value expresses the amount of information lost when the data are approximated with a model. Therefore, the best model will be the one that is closest to the probabilistic process that generated the data; in other words, the one with the lowest AIC value, as it approximates the data with the lowest information loss. Furthermore, R² represents a goodness of fit measure. Its value varies from 0.0 to 1.0, where higher values are preferable. It can be interpreted as the variance proportion in the dependent variable accounted for by the regression model^(^ 13 ^)^. In this sense, after comparing the spatial model with the non-spatial one, the one that had the highest R² value and the lowest AIC was chosen for analysis.

Microsoft Excel was used for calculating the mortality rates and producing the line graphs for descriptive statistics. Calculation of the local empirical Bayesian rate, GMI and LMI was performed using the TerraView 4.2.2 software, whereas the joinpoint regression analysis was performed in the Joinpoint Regression software, version 4.7.0.0. All maps were created using the QGIS software, version 2.4.17. The non-spatial OLS regression was performed using the Stata software, version 12, and the spatial GWR regression was done in the GWR software, version 4.0.9.

### Ethical aspects

As the study uses free-access secondary data available from the Unified Health System Information Systems, it waives prior approval from any Research Ethics Committee. However, it is important to emphasize the ethical commitment in data collection and analysis, as recommended in resolutions No. 466/12 and No. 510/16, so that no personally identifiable information, such as name or address, is present in the database.

## Results

Between 2010 and 2020 there were 601,521 deaths related to DM in Brazil. This number represents a mean mortality rate of 29.8 *per* 100,000 inhabitants.

In Table 1, it can be seen that the mortality rate among females (32.1 *per* 100,000 inhabitants) is higher than both the rate among males (26.9 *per* 100,000 inhabitants) and the overall rate observed in the country (29.8 *per* 100,000 inhabitants). In addition to that, the mortality rate increases with age, reaching its peak in the age group of 80 years old or more (508.46 *per* 100,000 inhabitants). Among the racial groups, the mortality rate is higher in individuals of mixed race (36.16 *per* 100,000 inhabitants) and, among the schooling levels, it is higher in people with up to three years of studies (59.53 *per* 100,000 inhabitants).

**Table 1 -:** Sociodemographic characterization of the death cases due to Diabetes *Mellitus* (*per*100,000 inhabitants) in Brazil, 2010-2020. Brazil, 2022

Variable	Total deaths (%)	Mortality rate*
	601,521 (100%)	29.8
Gender		
Female	331,150 (55.0%)	32.1
Male	270,553 (45.0%)	26.9
Age (years old)		
10-19	1,088 (0.1%)	0.32
20-29	3,976 (0.6%)	1.15
30-39	9,342 (1.5%)	2.87
40-49	26,887 (4.4%)	10.00
50-59	72,854 (12.1%)	34.49
60-69	136,522 (22.6%)	99.61
70-79	170,140 (28.2%)	230.82
>80	180,416 (29.9%)	508.46
Race/Skin color		
White	295,588 (49.1%)	31.85
Black	54,662 (9.0%)	33.00
Brown	218,790 (36.3%)	36.16
Asian	3,617 (0.6%)	24.26
Indigenous	1,149 (0.1%)	15.55
Schooling (years)		
0-3	291,229 (48.3%)	59.53
4-7	106,068 (17.6%)	23.78
8-11	59,248 (9.8%)	19.98
>12	20,207 (3.3%)	3.15

*Mortality rate *per* 100,000 inhabitants

In terms of the overall mean mortality rate in the Brazilian regions during the study period, the Northeast (34.4 *per* 100,000 inhabitants) and South (31.4 *per* 100,000 inhabitants) regions stand out with the highest rates, followed by Southeast (29.4 *per* 100,000 inhabitants), Midwest (23.7 *per* 100,000 inhabitants) and North (23.0 *per* 100,000 inhabitants). The time trend analysis evidenced a rise in overall mortality throughout the period, evidenced by the increasing behavior of the straight line.

The AAPC corresponding to the period was considered for the analysis of the mortality trends. In this way, it was possible to observe significant heterogeneity in the Brazilian territory, even in states from the same region. In the country as a whole, 11 states presented a significant increase in the mortality rate due to DM, whereas two states and DF showed a reduction. The remaining 13 districts did not present statistical significance, thus interpreting them as with a stable trend (Table 2).

The following states stand out in the North region: Amapá (AAPC: 6.1; 95% CI: 3.3 – 8.9) and Amazonas (AAPC: 5.8; 95% CI: 4.2 – 7.3), due to the significant increase in the mortality rate due to DM; similarly although less significant to the states of Maranhão (AAPC: 2.6; 1.5 – 3.7) and Alagoas (AAPC: 2.2; 95% CI: 0.9 – 3.5) in the Northeast, Minas Gerais (AAPC: 1.6; 95% CI: 0.6 – 2.5) in the Southeast, Rio Grande do Sul (AAPC: 3.9; 95% CI: 2.1 – 5.8) in the South, and Goiás (AAPC: 2.4; 95% CI: 1.6 – 3.3) in the Midwest. Regarding the reduction in the outcome incidence rates, the following states stand out: Ceará (AAPC: -2.2; 95% CI: -3.8 – -0.6) and Rio de Janeiro (AAPC: -1.3; 95% CI: -2.1 – -0.5), as well as *Distrito Federal* (AAPC: -0.3; 95% CI: -2.8 – 2.3), located in the Northeast, Southeast and Midwest of the country, respectively.

**Table 2 -:** Annual Percentage Change of mortality due to Diabetes *Mellitus* in Brazil, 2010-2020. Brazil, 2022

**State**	APC*1 **(95% IC** ^§^ **)**	**JP** ^†^	APC*2 **(95% IC** ^§^ **)**	**JP** ^†^	**AAPC** ^‡^ **(95% IC** ^§^ **)**
North Region					
Rondônia	1.3 (-1.1; 3.7)				1.3 (-1.1; 3.7)
Acre	1.9 (-1.0; 5.0)				1.9 (-1.0; 5.0)
Amazonas	5.8^||^ (4.3; 7.3)				5.8^||^ (4.3; 7.3)
Roraima	6.1^||^ (4.4; 7.9)	2017	-9.4 (-1.3; 0.5)		2.5^||^ (0.4; 4.5)
Pará	3.3^||^ (2.1; 4.4)				3.3^||^ (2.1; 4.4)
Amapá	6.1^||^ (3.3; 8.9)				6.1^||^ (3.3; 8.9)
Tocantins	4.1^||^ (1.8; 6.5)				4.1^||^ (1.8; 6.5)
Northeast Region					
Maranhão	2.6^||^ (1.5; 3.7)				2.6^||^ (1.5; 3.7)
Piauí	1.5^||^ (0.0; 2.9)				1.5^||^ (0.0; 2.9)
Ceará	-2.2^||^ (-3.8; -0.6)				-2.2^||^ (-3.8; -0.6)
Rio Grande do Norte	0.2 (-1.2; 1.6)				0.2 (-1.2; 1.6)
Paraíba	-0.8 (-1.8; 0.2)				-0.8 (-1.8; 0.2)
Pernambuco	-0.2 (-1.5; 1.2)				-0.2 (-1.5; 1.2)
Alagoas	2.2^||^ (0.9; 3.5)				2.2^||^ (0.9; 3.5)
Sergipe	-0.6 (-2.4; 1.2)				-0.6 (-2.4; 1.2)
Bahia	1.9^||^ (1.1; 2.6)				1.9^||^ (1.1; 2.6)
Southeast Region					
Minas Gerais	-0.4 (-1.8; 1.1)	2015	4.0^||^ (2.0; 6.1)		1.6^||^ (0.6; 2.5)
Espírito Santo	0.0 (-1.8; 1.8)				
Rio de Janeiro	-4.6^||^ (-6.2; -2.9)	2014	1.5^||^ (-0.2; 2.8)		-1.3^||^ (-2.1; -0.5)
São Paulo	-1.9 (-4.1; 0.4)	2014	2.2^||^ (0.6; 3.8)		0.4 (-0.7; 1.4)
South Region					
Paraná	0.4 (-1.1; 2.0)				0.4 (-1.1; 2.0)
Santa Catarina	1.3^||^ (0.5; 2.1)				
Rio Grande do Sul	0.1 (-2.8; 3.0)	2015	9.0^||^ (5.1; 13.1)		3.9^||^ (2.1; 5.8)
Midwest Region					
Mato Grosso do Sul	1.5 (-0.3; 3.4)				1.5 (-0.3; 3.4)
Mato Grosso	2.9^||^ (1.1; 4.7)				
Goiás	2.4^||^ (1.6; 3.3)				2.4^||^ (1.6; 3.3)
*Distrito Federal*	-5.4^||^ (-9.2; -1.4)	2015	6.4^||^ (0.7; 12.4)		-0.3^||^ (-2.8; 2.3)

^*^APC = Annual Percentage Change; ^†^JP = Joinpoint (year when the straight line segment changes); ^‡^AAPC = Average Annual Percentage Change; ^§^95% CI = 95% Confidence Interval; ^||^p<0.05

**Figure 1 -:**
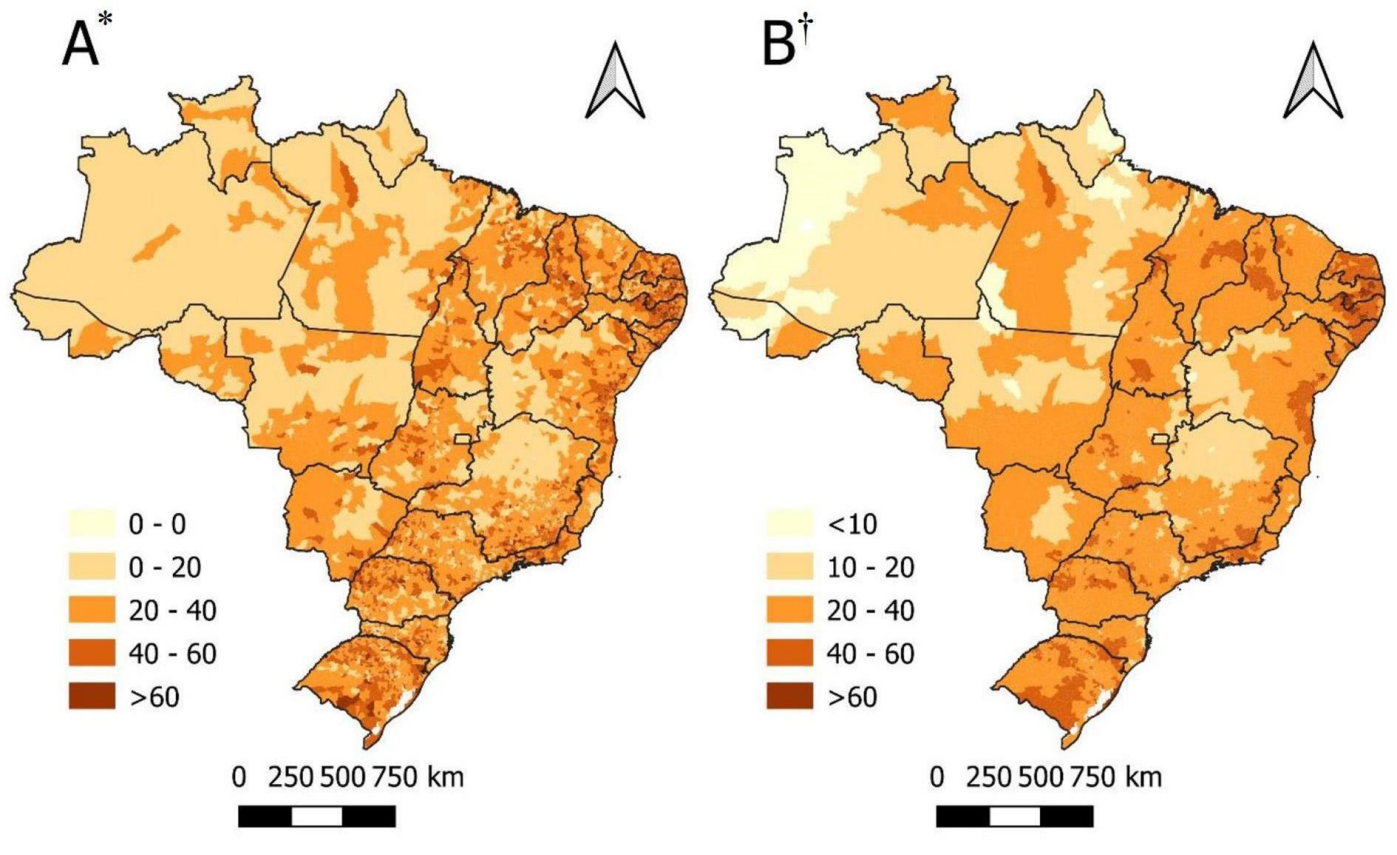

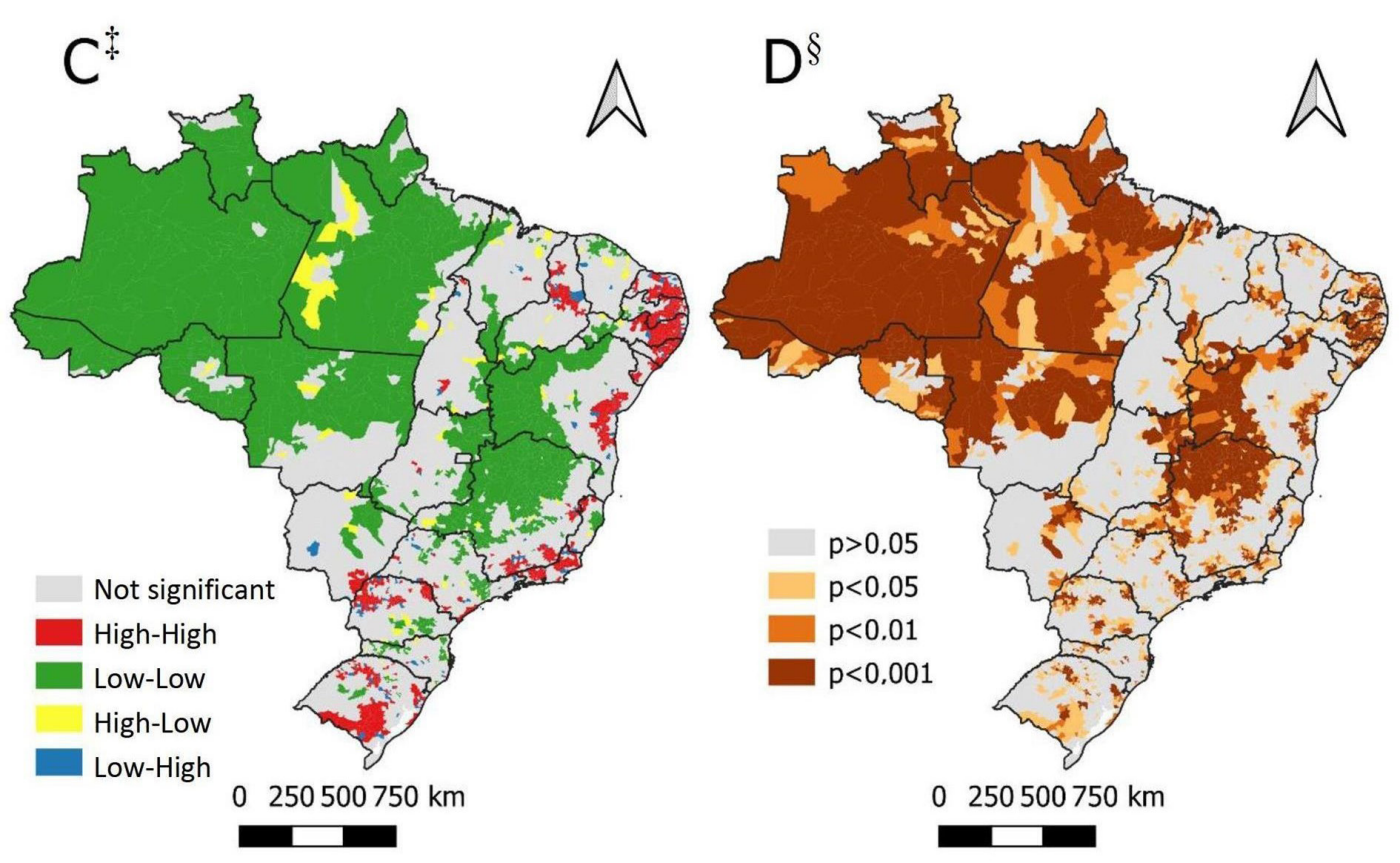
Spatial distribution and autocorrelation of mortality due to Diabetes *Mellitus* in Brazil during the 2010-2020 period, by municipality and *per* 100,000 inhabitants. Brazil, 2022

^*^A = Map of the unadjusted mortality rates per 100,000 inhabitants; ^†^B = Map of the mortality rates smoothed by means of the local empirical Bayesian method *per* 100,000 inhabitants. ^‡^C = Map of the global spatial autocorrelation of mortality due to Diabetes *Mellitus* in Brazil (LISA Map); ^§^D = Map of the local spatial autocorrelation of mortality due to Diabetes *Mellitus* in Brazil (mirror of the p-value, referring to the LISA Map)

Figure 1A displays the unadjusted values corresponding to the DM mortality rates in the Brazilian municipalities, which varied from zero to over 60 deaths *per* 100,000 inhabitants, heterogeneously distributed across the Brazilian regions. Additionally, Figure 1B represents the rates smoothed by means of the local empirical Bayesian method, more clearly highlighting the concentration of high mortality rates in the Northeast and South regions, with the states of Paraíba (PB) and Pernambuco (PE) standing out in the red areas. The lowest Bayesian rate was detected in Amaturá (AM) with 4.3/100,000 inhabitants and the highest, in Serraria (PB) with 78.0/100,000 inhabitants.

In addition, by means of the GMI, a positive autocorrelation was observed, I=0.46 (p=0.01), as well as presence of spatial clusters throughout Brazil. Consequently, the next step was to verify the spatial autocorrelation patterns by means of the LMI. Clusters of high-high patterns were observed in several municipalities throughout the country, especially in the Northeast Brazilian region, with emphasis on the states of Rio Grande do Norte, Paraíba, Pernambuco, Alagoas and Sergipe. Therefore, the states of Rio de Janeiro, Paraná and Rio Grande do Sul also presented high-high clusters that deserve emphasis. On the other hand, the low-low clusters were concentrated in the municipalities from the North region and across Minas Gerais, Bahia and Goiás (Figure 1C C). Significance and non-stationarity clusters were observed in the LISA Map (Figure 1D), revealing areas of statistically significant spatial dependence (p<0.05), with a particular emphasis on the low-low clusters and high-high clusters in the Northeast region (p<0.001).

After identifying the spatial pattern of deaths due to the disease, the relationship with sociodemographic, economic and health indicators was analyzed. Through the OLS regression, it was identified that the illiteracy rate among individuals over 18 years old, Gini index, *per capita* income, formalization degree among individuals over 18 years old, percentage of households with a bathroom and running water, percentage of households with a density greater than two, Family Health Strategy (FHS) coverage and the social vulnerability index presented a relationship with mortality due to DM at a significance level of p<0.20. Therefore, they were included in a multivariate OLS model with adjustment variables, as shown in Table 3. The model did not present multicollinearity, as the overall VIF of the model and the individual VIF of each variable were below 10.

In the univariate model, the *per capita* income, social vulnerability, illiteracy (>18 years old), Gini index, percentage of households with a bathroom and running water, percentage of households with a density greater than two and FHS coverage variables obtained a significant p-value (p<0.05), with the last five having a p-value of p<0.001. In the multivariate OLS model, the variables that were included remained significant, except for the Gini index (p=0.52), *per capita* income (p=0.44), and formalization degree in individuals >18 years old (p=0.36) (Table 3).

Furthermore, the multivariate model presented AIC = 45,094.4 and R² = 0.11. When the residuals of this regression were analyzed in terms of presence of spatial dependence, the results confirmed and indicated the need for geographical models. The same indicators from the OLS regression were tested in the geographical models, observing an improvement in the fit indices. The GWR model presented AIC = 43,092 and R² = 0.41, and was selected as the one that best described the variation of mortality and the one used in this study.

Through this model, it was verified that Gini index, social vulnerability index, and FHS coverage were the indicators that most influenced mortality dye to DM, with mean β values of 11.7, 9.05 and 3.9, respectively. In addition, the indicators regarding the illiteracy rate in individuals over 18 years old and the proportion of households with a density greater than two obtained β values very close to zero and should be interpreted with caution. *Per capita* income, formalization degree in individuals over 18 years old and social vulnerability index did not present significant values both in the non-spatial and spatial models, and should only be interpreted in relation to the local β of each municipality (Table 3).

**Table 3 -:** Ordinary Least Square (OLS) regression model corresponding to mortality due to Diabetes *Mellitus* in Brazil, 2010-2020. Brazil, 2022

	Univariate OLS*			Multivariate OLS*			GWR^†^	
	**Coefficient**	**Standard Error**	**p-value** ^‡^	**Coefficient**	**Standard Error**	**p-value** ^‡^	**Mean β** ^§^	**Standard Deviation**
Illiteracy in individuals >18 years old	0.01	0.002	<0.001		0.56	0.04	<0.001	0.20	0.68
Gini	-1.66	0.30	<0.001		2.54	4.00	0.525	11.67	36.81
*Per capita* income	-0.0002	0.00008	0.01		0.001	0.002	0.444	0.01	0.03
Formalization degree in individuals >18 years old	-0.001	0.001	0.065		0.02	0.02	0.358	-0.07	0.17
% of houses with a bathroom and running water	0.008	0.0009	<0.001		0.23	0.02	<0.001	0.24	0.25
% of houses with density >2	-0.18	0.001	<0.001		-0.18	0.03	<0.001	-0.31	0.41
MHDI^||^	-0.10	0.27	0.705						
FHS^¶^ coverage	0.64	0.08	<0.001		2.32	0.83	0.005	3.89	9.44
Social Vulnerability Index	-0.42	0.15	0.005		6.79	3.92	0.083	9.05	31.13
*Bolsa Família* transfer	0.0001	0.0002	0.521						
Percentage of people registered in *CADÚnico* ^**^ receiving *Bolsa Família*	-0.0009	0.001	0.371						
CONSTANT				1.66		3.29	0.615	7.48	32.70

^*^OLS = Ordinary Least Square; ^†^GWR = Geographically-Weighted Spatial Regression Model; ^‡^p-value: Significant p-value (p<0.05); ^§^β = Mean Beta value; ^||^MHDI = Municipal Human Development Index; ^¶^FHS = Family Health Strategy; ^**^ *CADÚnico* = *Cadastro* Único (Single Registry)

**Figure 2 -:**
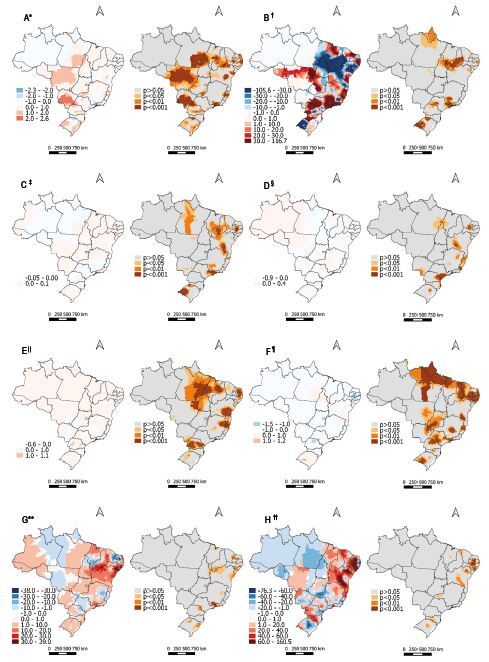
Spatial distribution via the regression corresponding to the relationship between socioeconomic indicators and mortality due to Diabetes *Mellitus* in Brazil during the 2010-2020 period, by municipality and *per* 100,000 inhabitants. Brazil, 2022

^*^A = Spatial distribution via regression of the relationship between the illiteracy rate among adults over 18 years old and mortality due to Diabetes *Mellitus* in Brazil; ^†^B = Spatial distribution via regression of the relationship between the Gini index and mortality due to Diabetes *Mellitus* in Brazil; ^‡^C = Spatial distribution via regression of the relationship between *per capita* income and mortality due to Diabetes *Mellitus* in Brazil; ^§^D = Spatial distribution via regression of the relationship between formalization degree in individuals over 18 years old and mortality due to Diabetes *Mellitus* in Brazil; ^||^E = Spatial distribution via regression of the relationship between the percentage of households with a bathroom and running water and mortality due to Diabetes *Mellitus* in Brazil; ^¶^F = Spatial distribution via regression of the relationship between the percentage of households with a density greater than two and mortality due to Diabetes *Mellitus* in Brazil; ^**^G = Spatial distribution via regression of the relationship between the Family Health Strategy coverage and mortality due to Diabetes *Mellitus* in Brazil; ^††^H = Spatial distribution via regression of the relationship between the social vulnerability index and mortality due to Diabetes *Mellitus* in Brazil

In consonance, Figure 2 outlines the thematic maps corresponding to the municipal estimates and the statistical significance of these indicators in mortality due to DM. The high illiteracy rate among individuals aged >18 years old exerted little influence on mortality due to FM at the global level; however, at the local level there was a significant and negative relationship in municipalities from Minas Gerais, Rio de Janeiro, São Paulo, Santa Catarina and Rio Grande do Sul; whereas municipalities from Pará, Mato Grosso, Mato Grosso do Sul, Minas Gerais, Espírito Santo, São Paulo and Rio Grande do Sul had a positive relationship (Figure 2A). The Gini index was the indicator that presented the greatest relationship with mortality due to DM, both at the global and local levels. It presented a negative relationship mainly in the states of Piauí, Maranhão and Rio Grande do Sul, whereas the positive relationship can be more clearly observed in São Paulo and Minas Gerais (Figure 2B).

*Per capita* income was the indicator that presented the lowest magnitude relationship with mortality due to DM, both at the global and local levels, and its results should be interpreted with caution. Consequently, a negative relationship is noted in municipalities from Pará and São Paulo and a positive one mainly in Piauí, Paraíba, Pernambuco, Bahia and Rio Grande do Sul (Figure 2C). Similarly, the formalization degree in people aged over 18 years old presented global and local values close to zero. In this regard, the negative relationship is observed more intensely in parts of Ceará, Paraíba, Pernambuco, Bahia and São Paulo, while the positive relationships are particularly evident in Espírito Santo and Rio de Janeiro (Figure 2D).

Furthermore, the proportion of households with a bathroom and running water exerted little influence on mortality due to DM, although it is worth noting that municipalities in Paraná showed a positive relationship with mortality (Figure 2E). The proportion of households with a density greater than two showed a negative relationship with mortality in the states of Paraíba, Pernambuco, Alagoas and Paraná, while the positive relationship can be observed more clearly in Rio Grande do Sul (Figure 2F).

On the other hand, the FHS coverage presented a strong relationship, both in global and in local values. Its local values showed a high influence on mortality due to DM, especially in the states of Ceará and Rio Grande do Norte, with a negative relationship, and in Alagoas and Sergipe, with a positive relationship (Figure 2G). Similarly, the social vulnerability index presented a strong association with mortality due to DM, both in global and in local values. In this regard, part of Paraíba and of Pernambuco with Rio Grande do Sul showed a weak and negative relationship, whereas part of Rio Grande do Norte, Paraíba, Pernambuco and Alagoas formed a band with a strong significant positive relationship (Figure 2H).

## Discussion

The study allowed identifying the Brazilian space-time pattern of mortality due to DM and its association with social development indicators. The states of Rio Grande do Norte, Paraíba, Pernambuco, Alagoas, Sergipe, Rio de Janeiro, Paraná and Rio Grande do Sul presented high-high clusters, with a particular emphasis on the Gini index and Family Health Strategy (FHS) coverage as social indicators with the greatest influence on the mortality rates due to the disease.

Mortality due to DM in the Brazilian regions represents a significant challenge to the Unified Health System (*Sistema Único de Saúde*, SUS) as well as to national socioeconomic development, highlighting the impact of the disease in the country, allied to its substantial contribution to the international panorama of DM. The overall situation is evidenced in other population-based time-analysis studies, with sociodemographic patterns similar to the findings in terms of gender, age group, race/skin color and schooling^(^ 14 ^-^ 16 ^)^.

In addition, it is worth noting that due to the demographic, social and economic heterogeneity found in Brazil, different patterns of mortality due to DM can be observed in various regions^(^ 3 ^)^, with the Northeast and South being marked by high-high values and the formation of high-risk clusters^(^ 17 ^)^. The prevalence of high mortality rates in these regions is influenced by factors related to biological conditions (high Body Mass Index), sociodemographic factors (low schooling levels, regional inequalities, barriers to access treatment, late diagnosis and other risk factors) and behavioral factors (sedentary lifestyle, unhealthy dietary patterns)^(^ 18 ^)^.

Furthermore, diverse evidence suggests that the greater the inequality in income distribution, the worse the health conditions in Brazil. This reinforces the significant socioeconomic gradient in the context of Chronic Non-Communicable Diseases (CNCDs), as reflected in the national^(^ 19 ^)^ and international^(^ 20 ^)^ scenarios, showing that the poorest populations, with higher social vulnerability levels and lower schooling, are the most affected by the loss in quality of life, increased years of life lost and deaths caused by DM^(^ 21 ^)^.

As an example, a statistically significant association was found between the illiteracy rate among adults over 18 years old, *per capita* income, Gini index and Human Development Index (HDI) variables and the outcome of death due to DM. With the adjustment of the multiple linear regression model, the poverty indicator remained statistically associated with the outcome, corroborating the literature^(^ 22 ^)^, especially in the North and Northeast Brazilian regions^(^ 3 ^,^ 18 ^)^. This finding indicates that increasing the employment and wage-earning opportunities can reduce the DM risk and, consequently, the number of deaths due to the disease.

Additionally, health indicators in individuals with CNCDs show poorer performance among beneficiaries of the *Bolsa Família* program since, to a greater extent, they present lower schooling levels, are of Black/Brown race and are concentrated in the North and Northeast regions. They also present higher incidence of obesity, multiple comorbidities, high cholesterol levels and alcohol and tobacco consumption, thus explaining the significant association of the variable with the outcome^(^ 23 ^-^ 24 ^)^.

Similarly, the global regions with low, low-average and average HDI values proved to be associated with a higher risk of deaths due to DM. This is because social determinants and conditions have repercussions on health disparities and are intertwined with social stratification, which interferes in the unequal distribution of power, prestige and resources^(^ 24 ^)^.

It is a fact that social vulnerability contributes to increased exposure and susceptibility to health risks and mortality, resulting from individual factors and collective conditions, as well as the availability of resources to address them. This is reflected in higher mortality due to DM in census tracts characterized by medium and high social vulnerability^(^ 21 ^)^. In this context, the importance of governmental transfers for the social protection of subjects in situations of vulnerability gains relevance.

Although the Brazilian health system was conceived to be universal, comprehensive and equitable for the entire population, its mixed institutional design with a significant percentage of health services provided by the private sector, seems to feed inequalities in access and unfavorable health outcomes, added to the inequality in regional income distribution^(^ 25 ^)^, representing an additional difficulty for the population with CNCDs living in poorer areas, as they face insufficient provision of services from the SUS.

The problem also seems to be related to access to health-related information. Health is a right for every Brazilian, and access to health information through education is a fundamental strategy for empowering individuals and communities, based on raising awareness about their health conditions and their role in this process^(^ 26 ^)^.

In this context, the contribution of Nursing can be enhanced through the use of methods that promote effective and meaningful learning, with planned and adapted educational interventions for each learning level^(^ 27 ^)^, such as discussion groups, the use of playful interventions, welcoming in the waiting room, campaigns and case studies, among others. In these opportunities, it is important to prioritize information about DM and healthy lifestyles^(^ 28 ^)^, in order to increase knowledge and skills for daily care, prevention of complications and unfavorable outcomes such as death.

It is noted that, in order to understand health outcomes, it is important to consider economic and social relationships, as well as their performance in geographic spaces and, consequently, their influence on the health-disease process of populations. In light of this, it is suggested to develop more in-depth studies involving population samples, field research studies and interventions, aiming to propose and implement measures targeted at changing this scenario.

It is reiterated that populations are subjected to cultural, demographic and socioeconomic heterogeneity, which leads to disparities in the quality of care provided, as well as in diagnostic capacity and in quality of the information provided; in addition, political and economic development also influences performance in health, extending to components such as education and income^(^ 18 ^)^.

A wide scope for overall improvement in the care of patients with DM can be seen ahead, with an urgent need to include the topic in the intra- and inter-sectoral public health policies developed in the country, especially in terms of reducing social disparities, expanding access to health and implementing policies for promotion, education, prevention and surveillance regarding diabetes^(^ 23 ^-^ 29 ^)^.

The study presents some limitations, namely: the use of secondary data collected for clinical purposes rather than research, with the possibility of presenting incomplete or inadequate information, combined with underreporting of deaths due to diabetes, as these cases are oftentimes recorded with acute or chronic events arising from the disease as their main cause, such as sepsis, cardiovascular diseases and vascular complications, among others. As a result, the findings point to the need to improve the quality of data collection related to the outcome, through raising awareness and providing training for healthcare professionals, in order to ensure robust databases and to improve accuracy of the epidemiological analyses, including the screening of mortality due the disease.

Another important limitation is the ecological fallacy resulting from casual inference in relation to the subjects, arising from the heterogeneous distribution of exposure to the factor under study and the other variables^(^ 30 ^)^.

Mortality due to DM in Brazil has increased in the last decade, particularly in the states from the Northeast and South regions, with a statistically significant association with income distribution indicators such as the Gini index and healthcare access indicators like the FHS coverage. Such results contribute to improving knowledge about the relationship between the socioeconomic and demographic indicators involved in mortality due to DM and the size of their effect on the death outcome.

## Conclusion

The space-time pattern of mortality due to Diabetes *Mellitus* in Brazil shows an overall increasing trend, where spatial clusters can be seen particularly in the Northeast and South regions, also revealing itself as associated with areas characterized by worse sociodemographic indicators such as income distribution, housing conditions, schooling and access to health.
